# Postgraduate Training Program in Pediatric Surgery: A Way Forward

**Published:** 2011-03-10

**Authors:** Jamshed Akhtar

**Affiliations:** Department of Pediatric Surgery, National Institute of Child Health Karachi, Pakistan

Postgraduate training is a dynamic activity. Over the years it is undergoing increasing standardization. In the past programs have been haphazard and inconsistent but now structured curriculum are in place. Comprehensive details related to criteria of entry, duration of training, core knowledge areas, and competencies required can be found. How program is to be run is explicitly chalked down and log books developed to be completed by trainees. In Pakistan one such model is implemented by College of Physicians and Surgeons Pakistan (CPSP). The program was developed through a well organized activity with explicit guidelines, under supervision of medical educationists, who initially trained program directors through various educational workshops. Few medical universities have also developed curriculum related to pediatric surgery but how they are developed is not known. While CPSP training program is uniform throughout the country, university program varies. There is also a comprehensive evaluation system with CPSP program and evaluation strategies are accessible to both trainees and trainers and evaluators.

The magic figure of three years of residency in pediatric surgery (after completing two years in general surgery with evaluation exam) in CPSP model is questioned by few program directors. In United States the total duration of training in pediatric surgery after general surgery residency, is two years. The 18 months training comprise of clinical pediatric surgery and the remaining six months may be spent in related clinical disciplines to enhance the educational experience, or get involved in scholarly activities. It raises question as to how general surgery residency in US model is beneficial to those seeking career in pediatric surgery. In this model the pediatric surgery resident spends only 25% of his total training in core curriculum of his own discipline. During this time period it is expected that trainee must document a total of 800 major pediatric surgery procedures as surgeon and the service providing training must cater for at least 1200 core surgical procedures per year. It is for pediatric surgeons to ponder for unique requirements for patient population and trainees [1 , 2].

The magic figure of three years of residency in pediatric surgery (after completing two years in general surgery with evaluation exam) in CPSP model is questioned by few program directors. In United States the total duration of training in pediatric surgery after general surgery residency, is two years. The 18 months training comprise of clinical pediatric surgery and the remaining six months may be spent in related clinical disciplines to enhance the educational experience, or get involved in scholarly activities. It raises question as to how general surgery residency in US model is beneficial to those seeking career in pediatric surgery. In this model the pediatric surgery resident spends only 25% of his total training in core curriculum of his own discipline. During this time period it is expected that trainee must document a total of 800 major pediatric surgery procedures as surgeon and the service providing training must cater for at least 1200 core surgical procedures per year. It is for pediatric surgeons to ponder for unique requirements for patient population and trainees [1 , 2].

Accreditation of the services for training in pediatric surgery is another painstaking assignment. An elaborate form has been developed by CPSP to be filled in by the program directors providing details of workload, facilities offered, number and expertise of teaching faculty etc. It is an observation that departments of pediatric surgery in the country are heterogeneous thus training imparted may not be uniform. Some services may get recognition even when facilities like ICU care, minimally invasive surgery etc are not optimal. Thus need of rotation to other centers where such exposure is guaranteed, must be looked into.

The two important areas not addressed with CPSP model are, compliance with the residency program, periodic evaluation and need of changes that may be required in this era of rapid technological advancement with new evidence based data related to diseases. No study has been conducted by pediatric surgeons in this regard to objectively address the issue. The Association of Paediatric Surgeons of Pakistan may gather a multi-institutional data to get some inference and suggest changes, if deemed necessary.

Another important issue is evaluation of trainees. In United States continuous periodic assessment is made and feedback given while in contemporary evaluation system with CPSP only end exam is the deciding point though residents do complete log books of their training and write dissertation or a scientific paper but these are not counted in the final examination. The training institutes thus play a passive role and may feel isolated. The input from supervisors must be acknowledged at some level for which integrity of trainers themselves is an issue. The role of trainers in residency program must be assessed periodically by respective institutes and monitoring body. In addition residents should be asked to write an annual confidential report of their supervisors. This two way accountability and evaluation helps in raising the standard of training program and evaluation of both trainees and trainers.

A residency program in pediatric surgery intends to impart advance knowledge coupled with hands on training to aspiring young doctors caring for children. An optimal program thus aims at producing safe surgeons, competent enough to take care of pediatric surgical patients referred to them.

## Footnotes

**Source of Support:** Nil

**Conflict of Interest:** None declared
